# Polyetheretherketone Cages Alone with Allograft for Three-Level Anterior Cervical Fusion

**DOI:** 10.5402/2012/452703

**Published:** 2012-02-01

**Authors:** Hong Liu, Avraam Ploumis, Chunde Li, Xiaodong Yi, Hong Li

**Affiliations:** ^1^Department of Orthopaedics, Peking University First Hospital, Beijing 100034, China; ^2^Division of Orthopaedics and Rehabilitation, Department of Surgery, Ioannina University Medical School, 45110 Ioannian, Greece

## Abstract

A total of 25 consecutive patients suffering from degenerative cervical disc disease who underwent three-level anterior cervical discectomy and fusion (ACDF) including polyetheretherketone (PEEK) cages packed with allograft were followed up for at least two years. The fusion rate reached 72% (18/25), and asymptomatic pseudarthrosis was seen in 6 patients but without mobility on flexion-extension radiographs, and revision surgery was not needed. Cage subsidence occurred at one level (C67), but it was not progressive, and reoperation was not necessary. A significant increase (*P* < 0.001) in fused segment angle (FSA) and fused segment height (FSH) was observed postoperatively. Similarly, a significant clinical improvement (*P* < 0.001) was demonstrated postoperatively in terms of Japanese Orthopedic Association (JOA) score and visual analog scales (VASs) score. PEEK cages alone with allograft proved to be a safe and effective surgical option in the treatment of three-level degenerative cervical disc disease. Although the fusion rate was not high, this technique may offer improvement of symptomatology and maintenance of cervical spine's sagittal profile.

## 1. Introduction

Various types of cages are currently widely used for the treatment of degenerative cervical disc disease [1]. However, controversy remains regarding a high incidence of complications, such as cage subsidence, kyphotic deformity, and pseudarthrosis [1–3]. Anterior cervical discectomy and fusion (ACDF) with plate fixation have been reported to reduce the above-mentioned complications [4–6]. Unfortunately, plate-related complications such as screw breakage, screw pullout, and esophagus perforation still exist though not very high [7–10]. Recently, good results have been reported regarding PEEK cages alone for treatment of one- or two-level ACDF [11–15]. In our institution, PEEK-cage-assisted ACDF was performed without additional plate fixation as a routine procedure since 2003, and we have achieved good clinical outcomes. Up to now, there are few reports demonstrating the fusion results after three-level PEEK cage implantation without additional plate fixation. Here, we reported 25 consecutive three-level ACDFs with PEEK cages alone. The fusion rate and radiographic and clinical outcomes are evaluated.

## 2. Methods

### 2.1. Study Population

From 2003 to 2008, 25 consecutive patients underwent three-level ACDF with PEEK cages in our institution. Surgical indication was unremitting radiculopathy, myelopathy, or a combination of both. Only patients with degenerative cervical disc disease were included. Patients with trauma, posterior longitudinal ligament (OPLL) or disc ossification, infections, or tumors were excluded. All patients were available for follow-up evaluation for more than 2 years after surgery. There were 14 males and 11 females. The ages ranged from 35 to 81 years, with a mean of 52.6 years (Table 1).

### 2.2. Surgical Technique and Postoperative Management

A standard Smith-Robinson procedure was used to approach the cervical spine anteriorly on the right side. After adequate decompression of the spinal cord and nerve roots, endplate cartilage was removed, but bony endplates were always preserved. The endplates were prepared for fusion by abrasion with a high-speed burr until they were matching the shape of the cage. Care was taken not to remove the bony endplate to prevent the subsidence of the cage. Intraoperative sizing of the cage using templates was carried out after distracting the disc space with the Caspar system. The key was not to overdistract the disc space in order to choose the appropriate size of cage. The cages (Solis, Stryker, Cestas, France) were packed with allograft (demineralized bone matrix, DBM, BaiAo, ShanXi, China) inside and then inserted into the disc space. Finally, the Caspar distractor was always compressed to remove any possible gap between the endplate and the cage and, thus, to provide ideal contact surface for fusion. The involved surgical levels were C3-4, C4-5, C5-6, and C6-7. All patients were immobilized postoperatively with a Philadelphia collar for 6 weeks.

### 2.3. Clinical Evaluation

Neck pain and arm pain were graded using a 10-point visual analog scale (VAS). Neurological outcomes were assessed using the Japanese Orthopaedic Association (JOA) score [16]. Surgical complications were also evaluated.

### 2.4. Radiographic Evaluation

Plain radiographs and flexion-extension films were taken preoperatively and at 2, 6, and 12 months after surgery. The following criteria were used for assessing radiographic success of fusion: (1) no lucent lines seen adjacent to endplates, (2) obliteration of disc space by bony trabeculae, (3) less than 2° of intervertebral motion or 2 mm of motion between the spinous processes at the operated segment on flexion-extension lateral radiographs [17, 18]. CT scans were used as a secondary measure when bridging trabecular bone was not observed or fusion was uncertain on plain radiographs [17–19].

The fused segment cervical lordosis (fused segment angle (FSA)) [6] was measured using the Cobb angle formed by the perpendicular line of the upper margin of the upper vertebral body and the one of the lower margin of the lower vertebral body of the fused segment.

The fused segment height (FSH) [6] was set as the distance between the midpoint of the upper margin of the upper vertebral body and the lower margin of the lower vertebral body of the fused segment. Cage subsidence was defined as greater than 3 mm reduction of FSH in the follow-up period.

Statistical analysis was performed with SPSS 13.0 software (SPSS Inc, Chicago, IL, USA), and statistical significance was defined as *P* < 0.05.

## 3. Results

### 3.1. Clinical Outcomes

The mean follow-up time was 25.6 months (range of 24–33 months). None of the patients suffered neurological deterioration after surgery. The mean ± SD JOA score before surgery, at 2 months after surgery, and at the final followup was 10.7 ± 2.17, 13.6 ± 1.47, and 14.1 ± 1.83, respectively. Similarly, mean ± SD VAS score before surgery, at 2 months after surgery, and at the final followup was 8.2 ± 1.23, 2.7 ± 0.96, and 1.9 ± 1.03, respectively. There was a significant difference in terms of both JOA and VAS scores before and after surgery or at the last followup (*P* < 0.001) (Table 2).

### 3.2. Radiographic Outcomes

**Figure 1:**
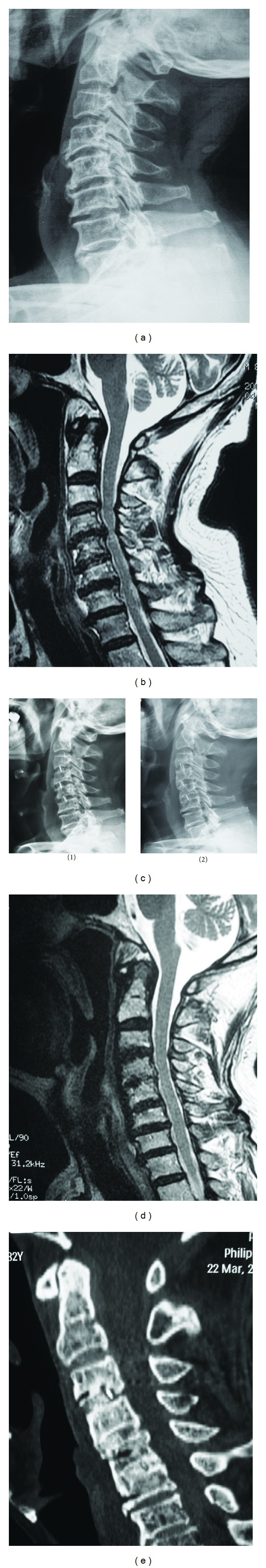
(a) Preoperative radiograph showing C3–C6 segment degeneration. (b) Preoperative sagittal T2W1 MR image revealed C34, C45, and C56 spinal cord compression. (c) Postoperative plain radiographs demonstrating adequate cervical lordosis and no cage subsidence 3 months (1) and one year (2) after ACDF. (d) Sagittal T2W1 MR image showing no spinal cord compression one year after ACDF. (e) Postoperative sagittal reconstruction CT image demonstrating C3–C6 fusion and no lucency between cages and endplates at one year postoperatively.

Radiographs of the cervical spine at the last followup revealed a solid fusion without signs of pseudoarthrosis in 18 cases with a fusion rate of 72% (Figure 1).

 The mean ± SD fused segment lordotic angle (FSA) was 1.3° ± 5.86 before surgery, 7.9° ± 3.62 at 2 months after surgery, and 7.0° ± 3.40 at the last follow-up examination. There was a significant increase in terms of FSA between preoperative and postoperative (at 2 months and at the last followup) measurements (*P* < 0.001) (Table 2).

 The mean ± SD fused segment height (FSH) was 71.3 ± 3.56 mm before surgery, 75.9 ± 4.12 mm at 2 months after surgery, and 75.3 ± 3.90 mm at the final follow-up examination. There was a significant increase in terms of the FSH between preoperative and postoperative (at 2 months and at the last followup) measurements (*P* < 0.001) (Table 2). 

### 3.3. Complications

There was one patient who had cerebral spinal fluid leakage, but he recovered in the first week following surgery. Two patients felt mild swallowing discomfort, but it disappeared within one month after surgery. Subcutaneous hematoma occurred in one patient due to obstructed drainage that was cleared two days postoperatively. Nonunion was seen in 6 asymptomatic patients at C34 and C56, respectively, but no motion instability was found on flexion-extension radiographs. Cage subsidence occurred at only one level (C67) in a patient with a C4–C7 fusion. Actually, the subsidence occurred immediately following the cage insertion into the disc space. Fortunately, no progressive cage migration was observed during the follow-up period, and reoperation was not necessary. No other surgical complications occurred, such as infection, cage migration or extrusion, and aggravating neurologic symptoms.

## 4. Discussion

Anterior cervical discectomy and fusion (ACDF) is currently the gold standard for surgical treatment of degenerative disc disease of the cervical spine [2, 3]. However, high-rate complications relating to tricortical iliac crest bone graft include pseudarthrosis, collapse, extrusion, subsidence, and resorption as well as donor site problems [12]. As a solution to these problems, various interbody fusion cages have been developed [1]. So far, cage-assisted ACDF has proven to be a safe and effective procedure for the treatment of degenerative disc disease [1–3, 5, 12, 14].

However, augmentation with plate fixation is usually needed, especially for multilevel ACDF [6, 19]. Although plate fixation has been found to increase the fusion rate, decrease cage subsidence rate, and thus maintain cervical lordosis, its inherent hardware complications consisting of screw pullout, screw breakage, and injury of esophagus still exist though not high [6–10]. In an attempt to solve the above-mentioned problems, PEEK cages alone for three-level ACDF were used at our institution. In our study, although the fusion rate was not as high as reported in the literature for cages with additional plating, good clinical results are still achieved [6, 11, 12, 19]. Cage subsidence rate was very low, and the cervical lordosis was well preserved at the final followup.

 The main reason for additional plate fixation in cage-assisted ACDF was the high cage subsidence rate in studies using cages without plating [1–3]. However, most of the studies included metal cages which had very different elasticity with bone and, consequently, led to high rate of cage subsidence [2, 4, 5]. Furthermore, another cause of cage subsidence might be the intraoperative overdistraction of the disc space and cage oversizing [12, 20]. A key point to prevent cage subsidence in our study was the avoidance of bony endplates removal (as it occurred in only one patient within the early practice of our study), but only abrasion of the cartilaginous portion was performed.

PEEK is a nonresorbable polyaromatic polymer with similar elasticity to bone [14]. The close match in the elastic modulus aids in minimizing cage subsidence and optimizing fusion rate that our study has revealed. Good clinical and radiographic results had been reported regarding PEEK cages alone for the treatment of one- or two-level ACDF [11–15]. Also, in a comparative study of efficacy of plate construct augmentation versus PEEK cage alone for one- or two-level ACDF, no significant clinical difference was found between groups [6]. To our experience, the presence of titanium spikes and retention teeth on the superior and inferior surfaces of the cage used in our study provides a secure fixation and prevents migration or extrusion of the cage. For this reason, PEEK cages alone without plate fixation can be used even for three-level cervical fusion without additional posterior fixation.

However, though our preliminary results are encouraging, good filling material is warranted to further improve the fusion rate, and long-term followup of the surgically treated cases is still needed. Moreover, prospective randomized and controlled clinical studies are warranted to compare the usefulness of PEEK cages alone and that of plate fixation and autograft for ACDF.

## 5. Conclusion

In summary, PEEK cages alone with allograft proved to be a safe and effective alternative in the treatment of three-level degenerative cervical disc disease. They may obtain clinical improvement, can maintain cervical spines sagittal profile, and obviate the complications related to iliac crest graft harvest and plate fixation.

## Figures and Tables

**Table 1:** Demographic data.

Mean age (years)	52.6 (range, 35–81)
Male	14
Female	11
C34, C45, C56	12
C45, C56, C67	13
Radiculopathy	9
Myelopathy	10
Radiculomyelopathy	6

**Table 2:** Clinical and radiographic results (mean ± SD).

	Preop	Postop 2 months	Postop 1 year
JOA	10.7 ± 2.17	13.6 ± 1.47	14.1 ± 1.83
VAS	8.2 ± 1.23	2.7 ± 0.96	1.9 ± 1.03
FSA	1.3 ± 5.86	7.9 ± 3.62	7.0 ± 3.40
FSH	71.3 ± 3.56	75.9 ± 4.12	75.3 ± 3.90

JOA: Japanese Orthopaedic Association; VAS: visual analog scale; FSA: fused segment angle; FSH: fused segment height.
